# Principles and procedures for handling out-of-domain and indeterminate results as part of ICH M7 recommended (Q)SAR analyses^☆^

**DOI:** 10.1016/j.yrtph.2018.12.007

**Published:** 2018-12-15

**Authors:** Alexander Amberg, Roxanne V. Andaya, Lennart T. Anger, Chris Barber, Lisa Beilke, Joel Bercu, Dave Bower, Alessandro Brigo, Zoryanna Cammerer, Kevin P. Cross, Laura Custer, Krista Dobo, Helga Gerets, Véronique Gervais, Susanne Glowienke, Stephen Gomez, Jacky Van Gompel, James Harvey, Catrin Hasselgren, Masamitsu Honma, Candice Johnson, Robert Jolly, Raymond Kemper, Michelle Kenyon, Naomi Kruhlak, Penny Leavitt, Scott Miller, Wolfgang Muster, Russell Naven, John Nicolette, Alexis Parenty, Mark Powley, Donald P. Quigley, M. Vijayaraj Reddy, Jennifer C. Sasaki, Lidiya Stavitskaya, Andrew Teasdale, Alejandra Trejo-Martin, Sandy Weiner, Dennie S. Welch, Angela White, Joerg Wichard, David Woolley, Glenn J. Myatt

**Affiliations:** a Sanofi, R&D Preclinical Safety Frankfurt, Industriepark Hoechst, D-65926, Frankfurt am Main, Germany; b Genentech, Inc., 1 DNA Way, South San Francisco, CA, 94080, USA; c Lhasa Limited, Leeds, UK; d Toxicology Solutions Inc., San Diego, CA, USA; e Gilead Sciences, 333 Lakeside Drive, Foster City, CA, USA; f Leadscope, Inc., 1393 Dublin Rd, Columbus, OH, 43215, USA; g Roche Pharmaceutical Research & Early Development, Pharmaceutical Sciences, Roche Innovation Center Basel, Switzerland; h Janssen Research & Development, 1400 McKean Road, Spring House, PA, 19477, USA; i Bristol-Myers Squibb, Drug Safety Evaluation, 1 Squibb Dr, New Brunswick, NJ, 08903, USA; j Pfizer Global Research & Development, 558 Eastern Point Road, Groton, CT, 06340, USA; k UCB Biopharma SPRL, Chemin du Foriest, B-1420, Braine-l’Alleud, Belgium; l Servier Group, Gidy, France; m Novartis Pharma AG, Pre-Clinical Safety, Werk Klybeck, CH-4057, Basel, Switzerland; n Consultant to Theravance Biopharma US, Inc., 901 Gateway Blvd, South San Francisco, CA, 94080, USA; o Janssen Pharmaceutical Companies of Johnson & Johnson, 2340, Beerse, Belgium; p GlaxoSmithKline, Park Road, Ware, Hertfordshire, SG12 0DP, UK; q National Institute of Health Sciences, Tokyo, Japan; r Toxicology Division, Eli Lilly and Company, Indianapolis, IN, USA; s Vertex Pharmaceuticals Inc., Discovery and Investigative Toxicology, 50 Northern Ave, Boston, MA, USA; t FDA Center for Drug Evaluation and Research, Silver Spring, MD, USA; u Takeda, Cambridge, MA, USA; v AbbVie Inc., North Chicago, IL, USA; w Merck Research Laboratories, West Point, PA, 19486, USA; x AstraZeneca, Macclesfield, Cheshire, UK; y Bayer Pharma AG, Investigational Toxicology, Muellerstr. 178, D-13353, Berlin, Germany; z ForthTox Limited, PO Box 13550, Linlithgow, EH49 7YU, UK

## Abstract

The International Council for Harmonization (ICH) M7 guideline describes a hazard assessment process for impurities that have the potential to be present in a drug substance or drug product. In the absence of adequate experimental bacterial mutagenicity data, (Q)SAR analysis may be used as a test to predict impurities’ DNA reactive (mutagenic) potential. However, in certain situations, (Q)SAR software is unable to generate a positive or negative prediction either because of conflicting information or because the impurity is outside the applicability domain of the model. Such results present challenges in generating an overall mutagenicity prediction and highlight the importance of performing a thorough expert review. The following paper reviews pharmaceutical and regulatory experiences handling such situations. The paper also presents an analysis of proprietary data to help understand the likelihood of misclassifying a mutagenic impurity as non-mutagenic based on different combinations of (Q)SAR results. This information may be taken into consideration when supporting the (Q)SAR results with an expert review, especially when out-of-domain results are generated during a (Q)SAR evaluation.

## 1. Introduction

In 2014, the International Council for Harmonization (ICH) issued their M7 guideline (“Assessment and control of DNA reactive (mutagenic) impurities in pharmaceuticals to limit potential carcinogenic risk”), which was revised in 2017 (ICH M7(R1), 2017). The guideline describes a hazard assessment process for impurities that reside or are reasonably likely to be present in a drug substance or product. In the absence of adequate experimental mutagenicity and/or carcinogenicity results, a structure-based computational toxicology or (Q)SAR^2^ analysis may be used as a test to predict DNA reactive (mutagenic) potential. (Q) SAR is a commonly used and relatively mature approach for predicting mutagenicity (Myatt et al., 2016). Based on the high predictive confidence levels (Dobo et al., 2012; Greene et al., 2015) and the cost of running such analysis relative to *in vitro* or *in vivo* studies, (Q)SAR assessments balance the need for a fast and efficient analysis while ensuring patient safety (Amberg et al., 2016). The (Q)SAR results, in turn, support the assignment of each impurity to one of five classes (shown in Table 1; Müller et al., 2006). This class assignment determines whether the impurity (1) requires no additional action, (2) requires additional laboratory testing, or (3) needs to be controlled below thresholds defined in the guideline.

The guideline recommends such (Q)SAR assessments to be based on the results from two complementary (Q)SAR methodologies: expert rule-based and statistical-based (ICH M7(R1), 2017, 2017; Myatt et al., 2016). The results from these models are combined, based on the “the absence of structural alerts” (ICH M7(R1), 2017), to generate an overall prediction to support the class assignment. That is, if both systems are non-alerting the class assignment is non-mutagenic.

(Q)SAR models use datasets of historical data as well as general scientific knowledge (such as structural alerts) from the literature based on known mechanisms of DNA reactive mutagenicity to generate a prediction. Since the models are based on what is known, they may not be able to predict with sufficient confidence a clear positive or negative outcome for novel chemicals. This may be due to conflicting evidence such that the influence of substituents on the reactivity of an alerting chemical moiety is not fully understood. These results will be referred to as indeterminate predictions in this publication (individual systems may refer to them as equivocal or other similar terms).

Another area where (Q)SAR models can present a challenge to users is when the structure being assessed falls outside the training or reference set used to generate the model. Such domain analysis is required as part of the (Q)SAR assessment since the ICH M7 guideline states that both methodologies should follow the general validation principles set forth by the Organization for Economic Co-operation and Development (OECD) (OECD, 2007). The third OECD validation principle requires the (Q)SAR model to assess whether each impurity is within the applicability domain of the model. (Netzeva et al., 2005; OECD, 2007; Carrió et al., 2014; Powley, 2015; Patlewicz et al., 2016). The applicability domain is generally defined as a region of chemical space within which a model makes predictions with a given reliability. It is often defined using structural features and/or properties of the training or reference set chemicals. Different modeling algorithms use distinct approaches to compute this applicability domain and therefore differ in coverage (Ellison et al., 2010; Chakravarti, 2012; Hanser et al., 2016; Myatt et al., 2016; Williams et al., 2016). A (Q)SAR model determines whether the impurity is outside the applicability domain of the model and such a result will be referred to as out-of-domain throughout this publication; however, different systems may use other terms such as not-in-domain).

The ICH M7 guideline describes that the (Q)SAR analysis may be supported by an expert review, especially in situations where the results are inconclusive (i.e., indeterminate or out-of-domain) as well as where there are valid reasons to overturn or refute a prediction. The standardized use of expert review has been detailed in several publications (Powley, 2015; Barber et al., 2015; Amberg et al., 2016) including as an *in silico* toxicology workflow that can be utilized under ICH M7 to generate predictions with improved accuracy that are consistent between different experts (Myatt et al., 2018).

The principles of the ICH M7 guideline are now routinely followed by the pharmaceutical industry and international regulatory agencies. Although the (Q)SAR assessment and expert review of the results has been discussed in a number of publications, there are some specific challenges associated with managing out-of-domain and indeterminate results, which have not been fully addressed. The following paper outlines current regulatory and industry approaches for handling out-of-domain and indeterminate results based on an industry survey. A series of case studies from regulatory submissions are provided to illustrate how out-of-domain or indeterminate results can be put into context. The paper also includes an assessment of the likelihood of misclassifying a mutagenic impurity as not mutagenic based on different combinations of (Q)SAR results (e.g., a negative expert rule-based result and an out-of-domain statistical-based result). How this information can be taken into consideration as part of an overall assessment is discussed.

## 2. Methodology

A general request was made to the pharmaceutical industry and regulatory authorities to outline current practices for handling out-of-domain and indeterminate results. This information was collated and summarized in Section 4 (Discussion).

To help understand the likelihood of misclassifying a mutagenic impurity as non-mutagenic based on different combinations of (Q)SAR results, a request was made to run the (Q)SAR models generally used for ICH M7 assessment over proprietary chemicals for which bacterial reverse mutation assay (Ames) data were available and provide a table containing the fields shown in Table 2. This included running different systems as detailed in the supplementary material.

The results were compiled into a single consolidated table for analysis. This involved a step to harmonize the results from different systems (including expert rule-based and statistical-based methodologies from Leadscope Inc. and Lhasa Limited) into the following calls for each methodology:

- **Positive:** A positive call (i.e., predicted to be mutagenic).
- **Negative:** A negative call that is within the applicability domain of the model (i.e., predicted to be non-mutagenic).
- **Indeterminate:** An indeterminate or equivocal call that is within the applicability domain.
- **Out-of-domain:** The (Q)SAR model considered the chemical outside the applicability domain.

In order to assess if additional model output that reflects the probability of a positive response can be used to support an overall assessment where the statistical-based model is out-of-domain, the following two situations are also considered when the system generates a probability score (such as the Leadscope Genetox Statistical Suite), as outlined in the supplemental material:

- **Out-of-domain with probability of being positive < 0.2:** The compound is considered outside the applicability domain of the statistical-based model; however, a probability score of less than 0.2 is generated
- **Out-of-domain with probability of being positive 0.2–0.4:** The compound is considered outside the applicability domain of the statistical-based model; however, a probability score of between 0.2 and 0.4 is generated

The rules used to harmonize the results across the different systems are included in the supplemental information.

## 3. Results

The following analysis of proprietary data was performed to help understand the likelihood of misclassifying a mutagenic impurity as not mutagenic (i.e., a false negative prediction) using different combinations of (Q)SAR results. The analysis is based on historical data from proprietary collections that include similar chemicals to those in a typical assessment of impurities such as low molecular weight chemicals used as starting materials and API (Active Pharmaceutical Ingredient)-like chemicals similar to the synthetic intermediates. The results were generated based on the methodology outlined in Section 2. The total number of chemicals considered was 15,886, which generally represents chemicals that were not used in building the models since (Q) SAR models for regulatory use are usually built using data from the public domain. The proportion of mutagenic compounds across the entire proprietary collection was 17.25%. It should be noted that no proprietary information was transferred as part of this process.

Table 3 shows the results where each model generates a positive or negative prediction and these predictions are inside the applicability domain of the models. The models used in this analysis are outlined in the supplementary material. The table includes the number of chemicals (“Count”) and the proportion of experimentally determined mutagenic compounds in each category. The total number of chemicals included in Table 3 is 10,083 out of the 15,886 chemicals analyzed (63.5%). When both predictions are positive approximately 60% of the chemicals are mutagenic, whereas when both are negative approximately 8% are mutagenic. When the results are not in agreement, the proportion of mutagenic compounds is between these two values.

Table 4 illustrates different situations when there is at least one out-of-domain result, which represents approximate 25% of cases in this study. The highest proportion of mutagenic compounds is when the expert rule-based model generates a positive or indeterminate result. When the statistical-based model is out-of-domain and the expert rule-based model is negative, there is a reduction in the proportion of mutagenic compounds identified. There is also a reduction when both models are out-of-domain. It should be noted that there were no examples in this study where the expert rule-based model result is out-of-domain and the statistical-based model is positive, negative or indeterminate.

Table 5 shows a more detailed analysis where the statistical-based model result is out-of-domain and the expert rule-based model result is negative. The table shows that the proportion of mutagenic compounds is lower when the statistical-based model generates a low probability score (less than 0.2) and no alerts are identified, even though the statistical-based model result is out-of-domain.

Table 6 summarizes different scenarios where there is an indeterminate call in one or both of the (Q)SAR methodologies which represents approximately 12% of examples in this study. The highest proportion of mutagens is shown when the expert rule-based model is positive and the statistical-based model is indeterminate, whereas the lowest proportion of mutagens is shown when the statistical-based model reports a negative and the rule-based model output is indeterminate. The percentage of mutagens for other scenarios is between these two values.

## 4. Discussion

### 4.1. Overview

Out-of-domain and/or indeterminate results are often encountered as part of an ICH M7 impurity assessment. This has been quantified, in part, through an analysis of new drugs approved in 2016 and 2017 that showed 18% of the impurities had an out-of-domain result (Powley, 2017). These out-of-domain and/or indeterminate results are often challenging for both pharmaceutical companies and regulatory agencies to generate an overall ICH M7 classification. Although a conservative approach would be to assume that indeterminate or out-of-domain (Q)SAR results are positive, this would compromise the desired utility of the computational analysis and could result in unnecessary additional drug development costs and delay the approval of new medicines.

A variety of approaches for handling out-of-domain and/or indeterminate results are being used across pharmaceutical companies as well as regulatory agencies to support an overall prediction (defined as “the absence of structural alerts” in the ICH M7 guideline). This is reflected in a further breakdown of drugs approved in 2016 and 2017 which quantified the different approaches for handling such situations including applying expert knowledge (for 70% of the submitted impurities), applying an additional model (for 6% of the submitted impurities), test/control (for 21% of the submitted impurities), and no follow-up (for 3% of the submitted impurities) (Powley, 2017). The following sections are based on the responses from the pharmaceutical industry and regulatory agencies as to how they handle out-of-domain and indeterminate results. This will cover different expert review strategies in addition to using another (Q)SAR model. The discussion will also review the results from the analysis of the likelihood of misclassifying a mutagenic impurity as non-mutagenic based on the different combinations of (Q)SAR results from the different methodologies.

### 4.2. (Q)SAR expert review

#### 4.2.1. Expert review definition and resulting actions

The application of expert knowledge has been shown to improve (Q) SAR predictive performance, particularly in resolving ambiguous outcomes such as out-of-domain results or indeterminate predictions, which is consistent with other published accounts (Dobo et al., 2012; Sutter et al., 2013). To support such an expert review, available computational methods generally provide information on the certainty of the prediction, such as a probability of a positive outcome. In addition, these methods often describe how the model generated the result. In the case of statistical-based methodologies, it is often possible to examine the training set and/or database analogs with detailed experimental data to understand how structural features or physicochemical properties influence the model’s prediction. For rule-based methodologies, it is often possible to inspect the structural features responsible for activation or deactivation of the alert along with an examination of plausible mechanisms, examples, and associated references for any activated alerts. Any expert review can make use of this information alongside the knowledge of the reviewer concerning DNA reactive mutagenicity, the quality of the experimental data, metabolism and knowledge derived from proprietary data (e.g., unpublished proprietary alerting chemicals).

To quantify the actions resulting from such a review, the US Food and Drug Administration’s (FDA) Center for Drug Evaluation and Research (CDER) between May 2016 and April 2017 analyzed 519 impurities for bacterial mutation using software from Leadscope, (2017), Lhasa Limited (Lhasa, 2017), and MultiCASE (MultiCASE, 2017). (Kruhlak et al., 2017) The expert-reviewed predictions were concordant with the consensus (Q)SAR results 87% of the time with:

- 2.1% of the negative consensus predictions changed to positive after the expert review
- 4.2% of the positive consensus predictions changed to negative after the expert review
- 61% of the indeterminate consensus predictions changed to negative after the expert review
- 11% of the indeterminate consensus predictions changed to positive after the expert review
- 28% of the indeterminate consensus predictions were not changed

These results (i.e., 4.2% of the positive consensus predictions changed to negative after the expert review) support the observation that the ICH M7 classification paradigm (that any positive prediction yields a positive overall call) risks generating a number of false positive predictions that can be corrected through the application of expert knowledge. Furthermore, it supports the conclusion that the majority of indeterminate predictions are not meaningful signals and can be readily downgraded to negative through a review of the model output and supporting analogs from the public domain.

#### 4.2.2. Use of analogs in an expert review

Structurally and/or toxicologically meaningful non-mutagenic analog(s) from public or proprietary databases or chemicals related to the impurity (such as the API or synthetic intermediate) are often used to support an overall prediction for an impurity when additional evidence is needed. This approach is sometimes referred to as read-across (Ball et al., 2016). As part of conducting a read-across assessment, the adequacy of the experimental design and results for the analog(s) are evaluated, as reviewed in Amberg et al. (2016). Specific analogs are often selected that cover any structural features that the model(s) identified as being potentially reactive (ICH M7(R1), 2017). It is valid and usual practice to discount mutagenic analogs when they contain one or more additional structural moieties that are more likely responsible for the mutagenic result.

The following examples illustrate expert reviews of out-of-domain results based on structural analogs.

##### Case study 1 (Impurity A) - Expert review based on analogs:

**Fig. 1.**
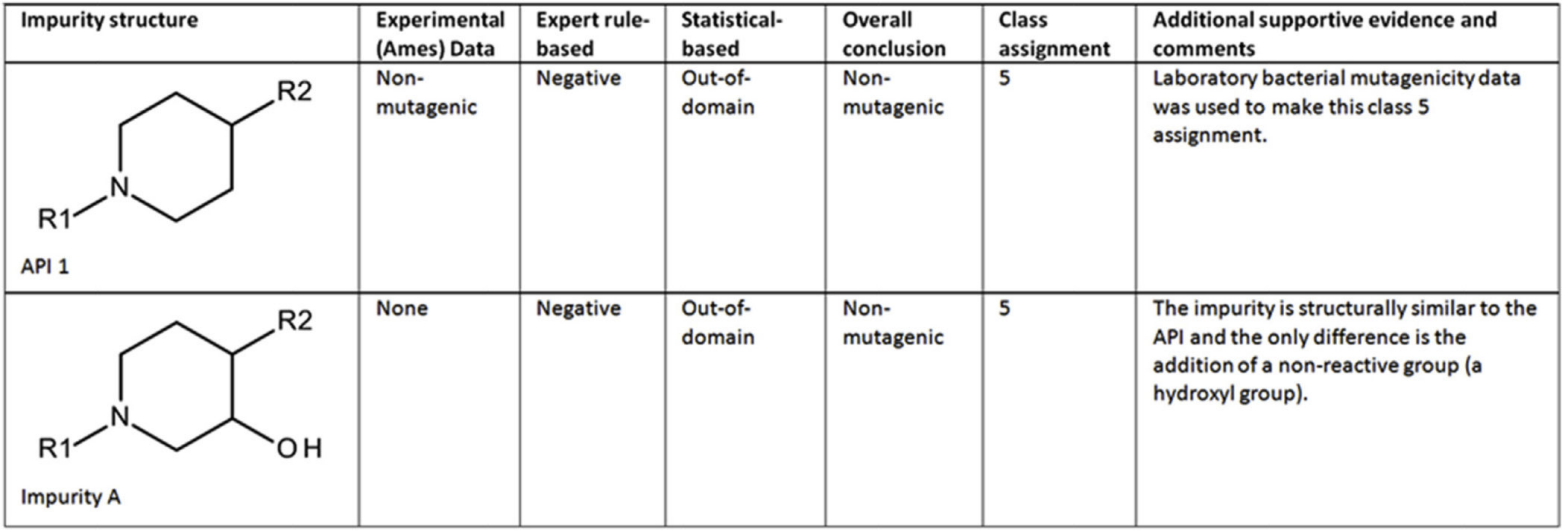
(Q)SAR assessment of impurity A.

An impurity (shown in Fig. 1) was predicted to be negative by an expert rule-based model. The impurity was out-of-domain in a statistical-based model as the compound contained a fragment not present in any chemicals in the training set and no nearest neighbors were identified in the model. The impurity is structurally similar to the API, which was experimentally determined to be non-mutagenic. The prediction results for both the impurity and the API were identical, and the API was also predicted to be out-of-domain by the statistical model for the same reasons as the impurity. Since the impurity is structurally similar to the API and the only difference is the addition of a non-reactive group (a hydroxyl group), the overall prediction is non-mutagenic and the impurity is assigned to class 5. A class 4 assignment was not used in this situation since neither the API nor the impurity share an alert associated with mutagenicity (i.e., the out-of-domain fragment was not considered an alert for mutagenicity). Some sponsors may consider this a class 4 compound to highlight that structural comparison with a known non-mutagenic analog has been performed. The US FDA interprets this situation as a class 5.

##### Case study 2 (Impurities B and C) - Expert review based on analogs:

**Fig. 2.**
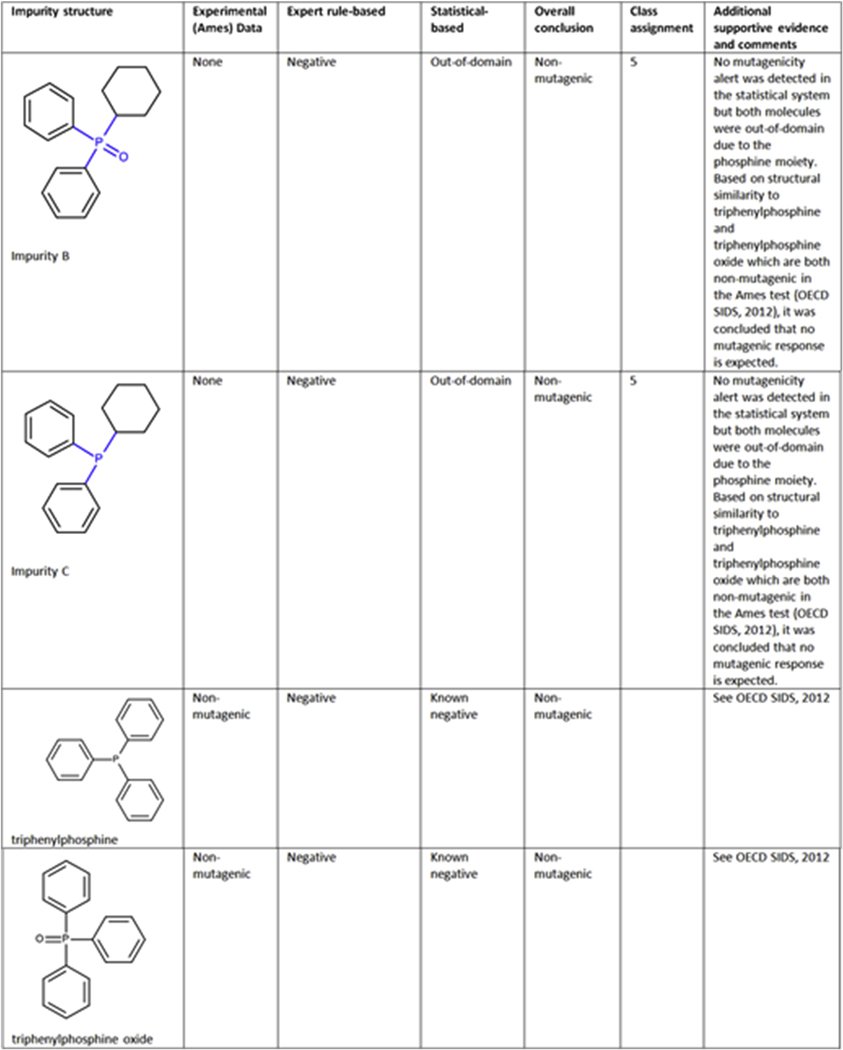
(Q)SAR assessment of impurities B and C.

Cyclohexyldiphenylphosphine oxide (Impurity B; CAS 13689–20-8) and cyclohexyldiphenylphosphine (Impurity C; CAS 6372–42-5) are impurities (shown in Fig. 2) occurring in the synthesis of a drug substance. Neither showed structural concern for mutagenicity using the expert rule-based model and were considered within the applicability domain. The statistical-based model predicted them as negative but both molecules were out-of-domain due to the phosphine moiety (highlighted in blue). Based on the structural similarity to triphenylphosphine (CAS 603–35-0) and triphenylphosphine oxide (CAS 791–28-6), which are both non-mutagenic in an Ames test (OECD SIDS, 2012), impurities B and C were concluded to be non-mutagenic (class 5).

#### 4.2.3. Assessment of non-reactive groups

For out-of-domain or indeterminate (Q)SAR results, additional supporting analysis to confirm that the impurity lacks any DNA-reactive potential has also been used (Powley, 2015). This includes a visual assessment of the compound to assure the lack of valid DNA-reactive alerts with plausible mechanisms, taking into consideration any unique alerts from proprietary information (Amberg et al., 2016) or knowledge of metabolic activation. This assessment often takes into consideration the strength of other model result(s) since models engineered under statistical-based or expert rule-based algorithms predict mutagenicity and consider applicability domain in different ways, and when one model is deficient, the other may be reliably used to make up for the deficiencies. Such an assessment may be supported by an inspection of the (Q)SAR model output when such reports visually illustrate a lack of potentially reactive features.

In addition, specific searches for significant functional groups and other substructures present in the impurity (performed manually or automated by a software application) against a database containing mutagenicity data (including proprietary data) is often performed. This may indicate that the impurity contains a potentially reactive group when the results contain significantly more positive examples linked to that particular substructure than would be expected by chance (i.e., enrichment of positives over background rate). Additionally, the (Q) SAR model may be applied to any substructure of the impurity to help determine the reactive potential for the components, when the whole chemical is out-of-domain.

There are several substructures, such as protection groups (e.g., tertbutyloxycarbonyl, or BOC), where their presence within an impurity may change the prediction from negative to out-of-domain. In these cases, such substructures are specifically known to block that portion of the molecule from chemical reactivity (Amberg et al., 2016). Therefore, the (Q)SAR model could be run on the substructure without the BOC group instead (i.e., on the free amine in the case of a BOC-protected-mine) and this resulting prediction used as part of an expert review.

##### Case study 3 (Impurity D) - Expert review based on an analysis of potentially reactive features:

**Fig. 3.**
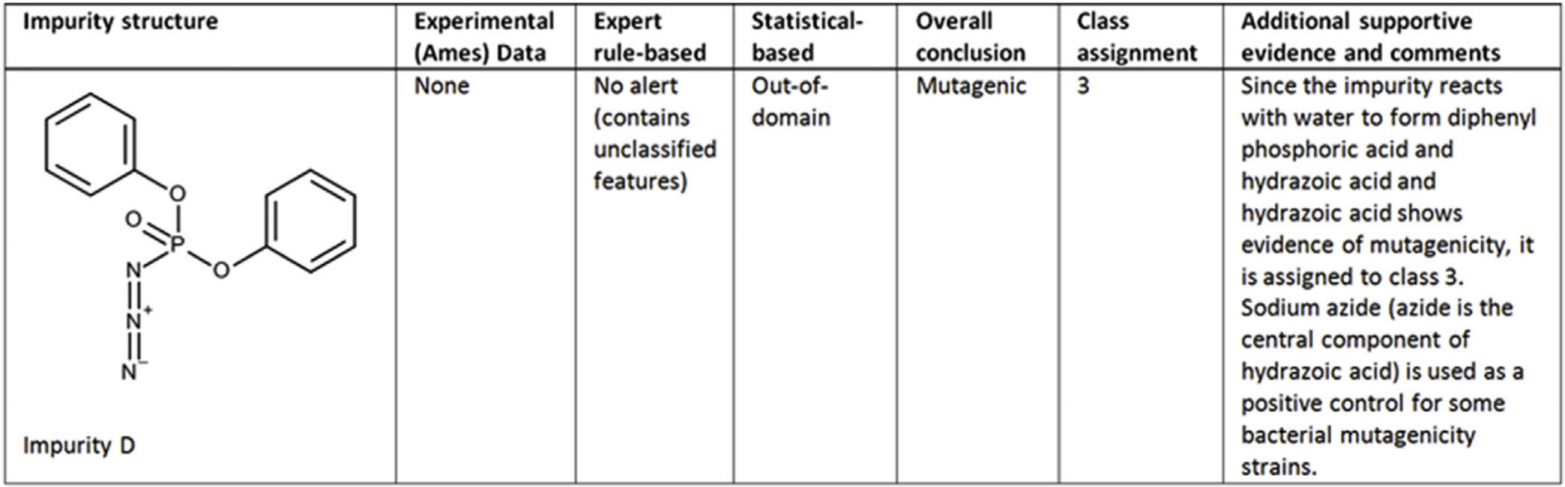
(Q)SAR assessment of impurity D.

As shown in Fig. 3, the rule-based expert system identified no alert but determined one or more features were present in the impurity that were not found in the reference set. Therefore, it is assigned as negative; however, there is uncertainty since it contains unclassified features. The statistical-based model determined the impurity is out-of-domain. It is known that the impurity reacts with water to form diphenyl phosphoric acid (838–85-7) and hydrazoic acid (7782–79-8). Since hydrazoic acid shows evidence of mutagenicity, a conservative action would be to assigned it to class 3; however, since (Sodium) Azide is negative in a 2-year cancer bioassay (NTP, 1991) it could be assigned as class 5 with appropriate justification.

##### Case study 4 (Impurity E) - Expert review based on features not covered:

**Fig. 4.**
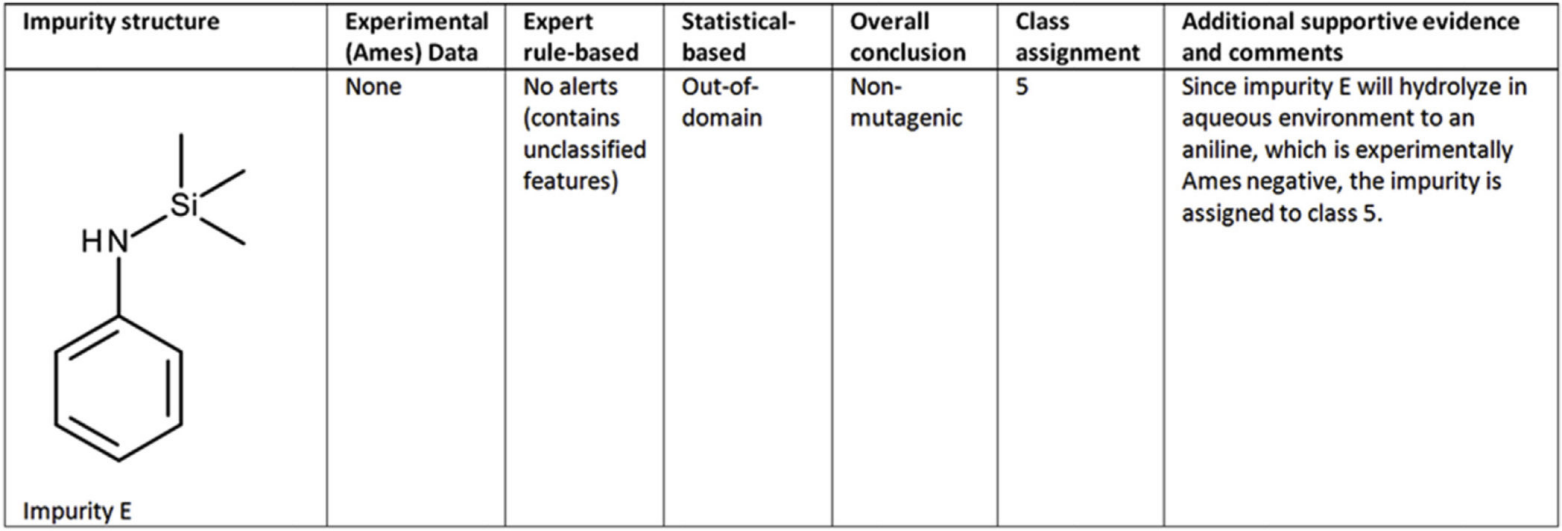
(Q)SAR assessment of impurity E.

As shown in Fig. 4, no alerts were identified using the expert rule-based system; however, it was determined to contain structural features not present in any of the reference set chemicals. Therefore, it was assigned as negative; however, there is uncertainty since it contains unclassified features. The statistical-based system also generated an out-of-domain call. Since impurity E will rapidly hydrolyze in an aqueous environment to the aniline, which is experimentally Ames negative (aniline is a publicly known non-mutagenic compound in Salmonella and *E. coli* (NTP, 1980).), the impurity is assigned to class 5.

#### 4.2.4. Situations when (Q)SAR methodology uses sub-models, i.e., GC versus AT primary reversion site

Some (Q)SAR systems include a battery of models including those for the traditional Ames (i.e., four strains to detect GC base pairs at the primary site of reversion) and an additional model for the AT base pair reversion site (i.e., *E*. *coli* WP2 or *E. coli* WP2 uvrA, or *E. coli* WP2 uvrA (pKM101), or *S. typhimurium* TA102). The database of compounds used to build the model for the GC base pair mutations is typically larger than that used for the AT reversion site. Therefore, it is more likely that a compound will be out-of-domain in the model for the AT reversion site. A prediction just at the GC primary reversion site may be sufficient to support a valid prediction in many cases. However, where the impurities contain specific AT alerting fragment(s), such as oxidizing mutagens, cross-linking agents, and hydrazines, the four strains in the traditional Ames would not be able to detect the mutagenicity of an impurity. In this case, further interrogation of the impurity in strains that detect the AT base pair reversion site may be warranted.

#### 4.2.5. Specific consideration for expert review of indeterminate (Q)SAR

Strategies for handling both indeterminate predictions and out-of-domain results are similar. One of the biggest differences is that there is a supporting dataset for the indeterminate prediction but it falls in the middle of the positive and negative predictive space. One effective strategy can be to review the training set for secondary features not contained in the impurity that could skew the prediction towards indeterminate from either positive or negative. Also, a lack of similarity of the impurity to the underlying training set chemicals can be used to overrule such a call. For example, a statistical-based model prediction of a high molecular weight impurity containing a hindered epoxide was indeterminate; however, an inspection of the training set indicated that the majority of the training set compounds responsible for the indeterminate call were unhindered and hence the prediction may be overruled.

### 4.3. Using an additional model

Although multiple available models may be built from the same or similar public databases, different modeling techniques, as well as methods for assessing the applicability domain, may give different results. For example, a new model may generate a similar result that is within the applicability domain, whereas the initial model’s result was out-of-domain.

In addition to using another public model directly, an alternative is to enhance an existing model through inclusion of proprietary structure (s) to increase the domain of the original model without substantially changing the original model. This has been particularly useful when many related compounds are out-of-domain and the expansion of the model includes one or more chemicals (e.g., API or key Ames tested intermediate) that are structurally related to the impurities. The addition of these structures is often sufficient to bring the impurity within the applicability domain but might change the probability score (or equivalent confidence score) and, in limited situations, the prediction of mutagenic potential for the impurity. Another approach is to create a new model using a training set built either exclusively from proprietary data or proprietary data combined with publicly available data (Jolly et al., 2015). Such modified models may need additional documentation describing the specific modifications (such as the chemicals added), as well as evidence that the revised model is consistent with the OECD’s (Q)SAR validation principles (OECD, 2007).

#### Case study 5 (Impurity F) - Assessment based on running an addition model and an expert review of analogs:

**Fig. 5.**
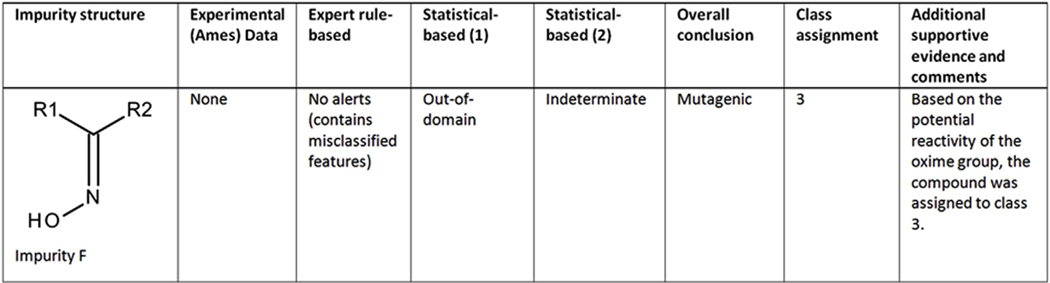
(Q)SAR assessment of impurity F.

For the impurity shown in Fig. 5, an expert rule-based model did not identify any alerts but flagged it as containing a structural feature shared with an experimentally determined mutagenic analog. A statistical-based model indicated the compound was out-of-domain. A second statistical-based model predicted the compound as indeterminate, highlighting the oxime group as a potentially reactive fragment. An examination of the structural analogs supporting the oxime group as a potentially reactive fragment showed that the examples most closely-related to Impurity F were mutagenic. In addition, two analogs were identified based on a substructure search of supplemental databases for the oxime group and both were experimentally determined mutagens. As a result of the potential reactivity of the oxime group, the impurity was assigned to class 3.

#### Case study 6 (Impurity G) - An assessment based on running a third model and expert review of analogs:

**Fig. 6.**
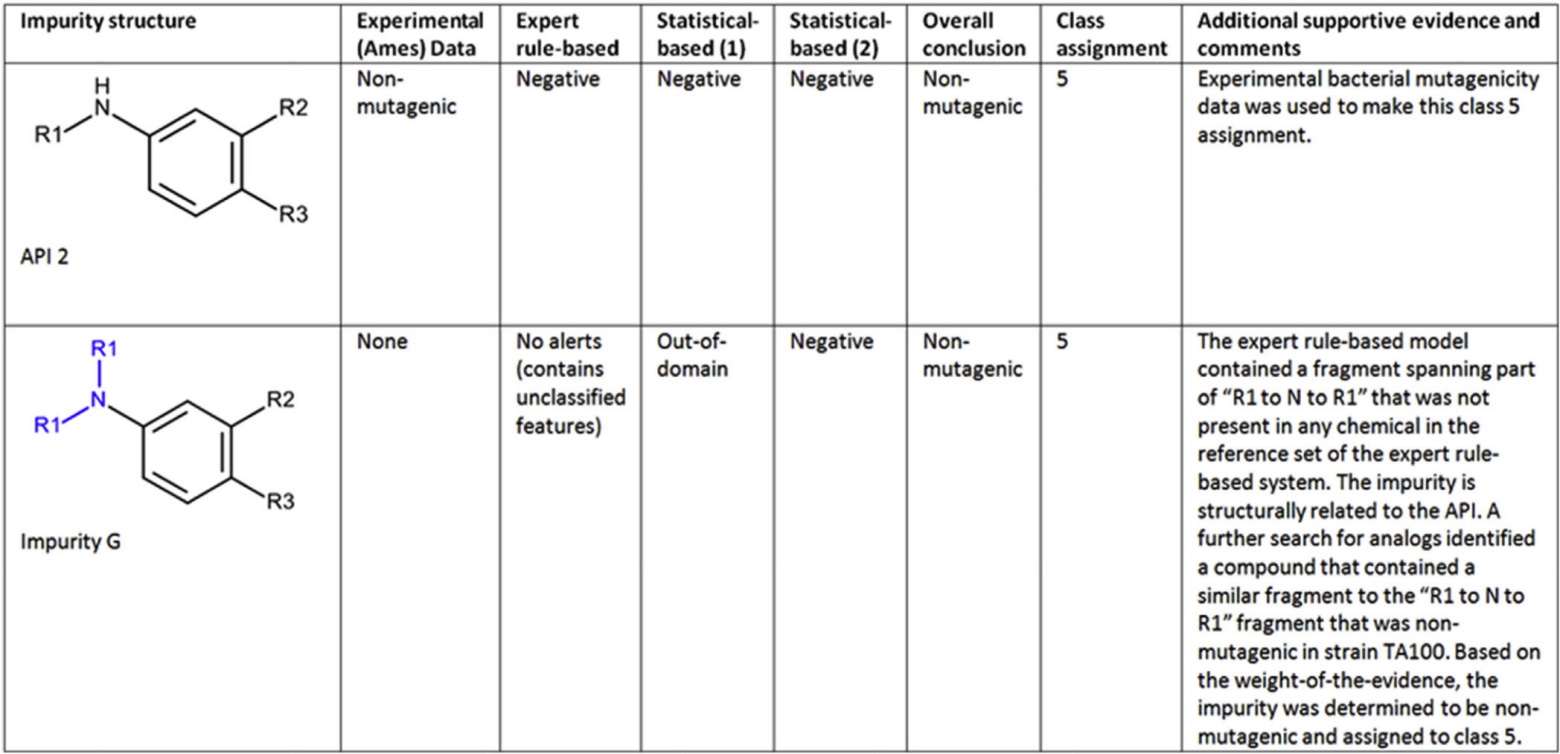
(Q)SAR assessment of impurity G.

An impurity (shown in Fig. 6) was predicted to be negative by an expert rule-based model; however, it contained a fragment spanning part of the “R1 to N to R1” (highlighted in blue) that was not present in any chemical in the reference set of the expert rule-based system. The statistical-based model determined the compound to be out-of-domain. A second statistical-based system predicted the impurity to be negative. The impurity is structurally related to the API (API 2), which was predicted negative (and in domain) for all three models run, and known to be experimentally negative. A further search for analogs identified a compound that contained a similar fragment to the “R1 to N to R1” fragment (highlighted in blue) that was negative in strain TA100. Based on the weight-of-the-evidence, the impurity was determined to be non-mutagenic and assigned to class 5.

#### Case study 7 (Impurity H) - Assessment based on running an additional model and expert review of an analog (API):

**Fig. 7.**
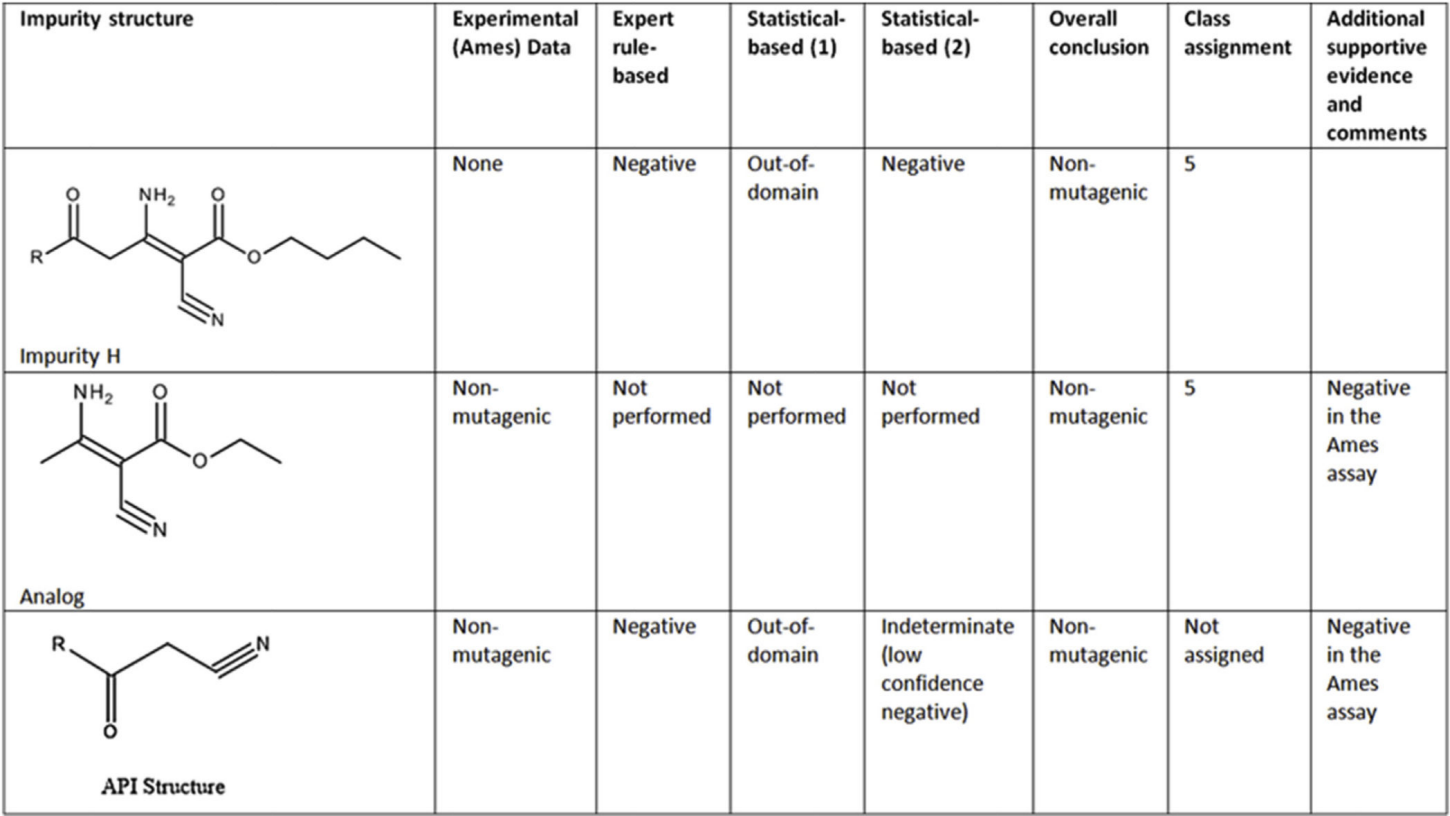
(Q)SAR assessment of impurity H.

This impurity (shown in Fig. 7) was negative in an expert rule-based model and out-of-domain in a statistical-based model. A second statistical-based model was run and it was determined to be negative and within the applicability domain of the model. The API was negative in the Ames test, predicted negative in the expert rule-based model and out-of-domain in a statistical model with a second statistical model providing an indeterminate call due to a low confidence negative prediction. Further assessment was made that the substituent on the impurity that was not contained in the parent was qualified by a negative Ames test on the same substructure (analog) with a similar environment. Hence, the impurity is assigned to class 5.

### 4.4. Class assignments - test or control to TTC?

Any situation where one or more of the results are out-of-domain or indeterminate, in general, requires an expert review to provide support for an overall negative prediction (as outlined previously). In the absence of any supportive evidence for out-of-domain or indeterminate call(s) (e.g., there are no adequate non-mutagenic analogs or it is not possible to verify that certain structural features of the impurity are not reactive or another model generates an out-of-domain call), the prediction would be considered uncertain and an Ames test may be prudent to make a final conclusion. Alternatively, it can be treated as mutagenic (class 3) and controlled to the Threshold of Toxicological Concern (TTC) defined in the ICH M7 guideline. In situations where there is sufficient supportive evidence and any positive signals from the models are refuted as part of an expert review, the impurity is generally assigned to class 5 or class 4 if any present alert is shared with an empirically non-mutagenic chemical such as an API.

### 4.5. Regulatory review – US FDA experience

ICH M7 submissions are handled by the individual review divisions at US FDA/CDER. The reviewers assess the information provided by the pharmaceutical applicant, including information on the software and models used, the results from the software, the overall conclusions and any associated expert review documentation for consistency with the ICH M7 guideline and to ensure the results and expert review are valid. In cases where the reviewer has questions or concerns, the (Q)SAR submission is provided to FDA/CDER’s internal Computational Toxicology Consulting Service (CTCS) for evaluation (Rouse et al., 2017). It should be noted that the reviewer will not re-run the predictions, but the Computational Toxicology Consultation Service staff may. Examples of such situations are:

1. poorly documented evaluations, unfamiliar software, software that does not allow for prediction interpretation consistent with ICH M7, or models that are not compliant with the OECD validation principles
2. situations when only a single methodology was used or only read-across (Ball et al., 2016) was used
3. when the overall conclusions conflict with the individual model predictions, without an explanation
4. when the most recent version of the software was not run and a change in the prediction is anticipated

In 2016, 217 consultation requests (for a total of 473 chemicals) were related to ICH M7 submissions, with 90% for generic drug applications and 10% for new drug applications (Kruhlak et al., 2017). In cases where the US FDA/CDER performs an independent (Q)SAR assessment of the impurities, this includes:

1. a review of the (Q)SAR data submitted
2. a further structure-based search (using exact, substructure and similarity-based searches) for additional experimental data on the impurities or any analogs
3. an independent (Q)SAR assessment using models from Leadscope Inc. (Leadscope, 2017), Lhasa Limited (Lhasa, 2017), and MultiCASE Inc. (MultiCASE, 2017)
4. an expert review of the results and related literature

Section 4.2.1. provides some additional information on performing an expert review at the US FDA.

### 4.6. Analysis of the likelihood of misclassifying a mutagenic impurity as non-mutagenic

**Fig. 8.**
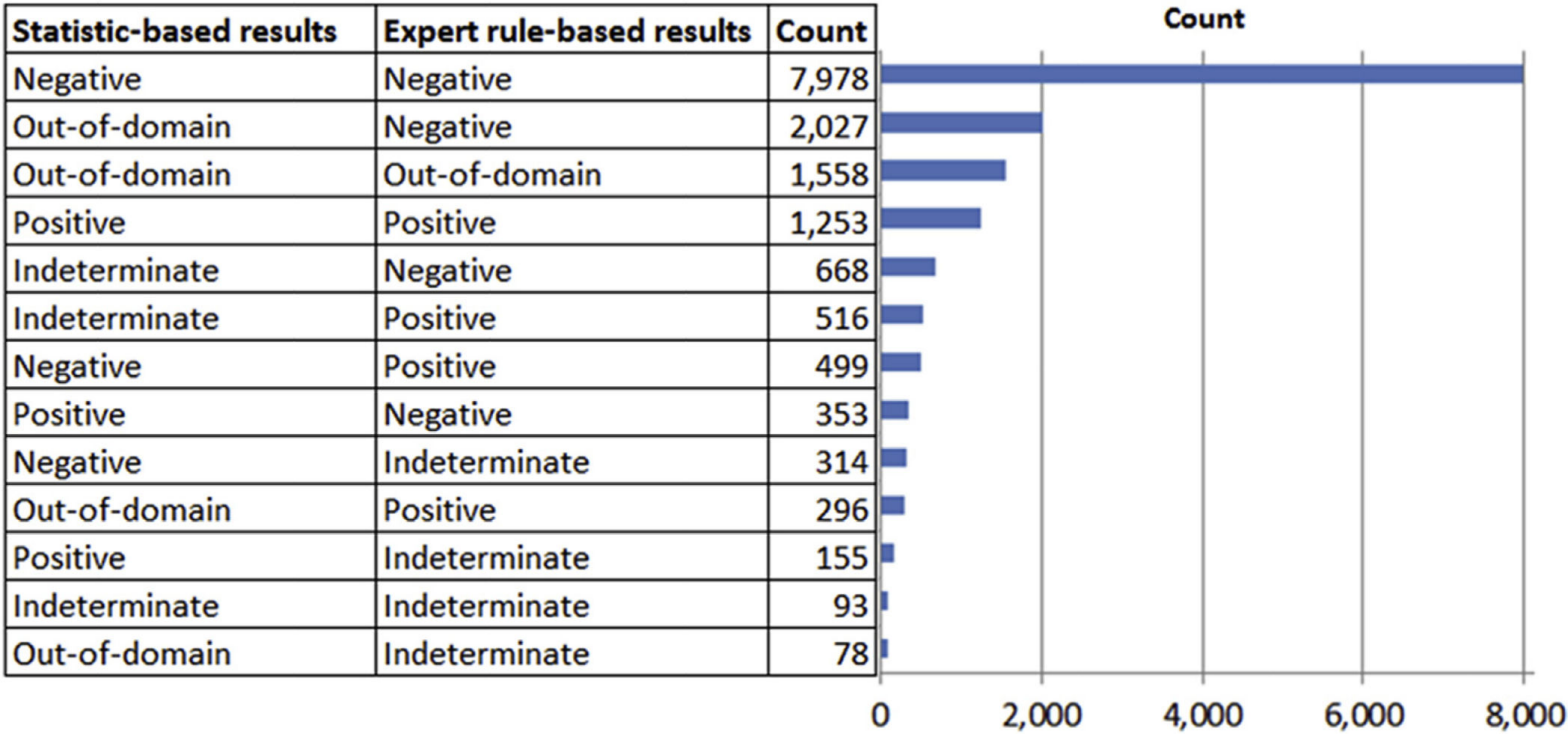
Illustration of the number of times different (Q)SAR results are encountered.

**Fig. 9.**
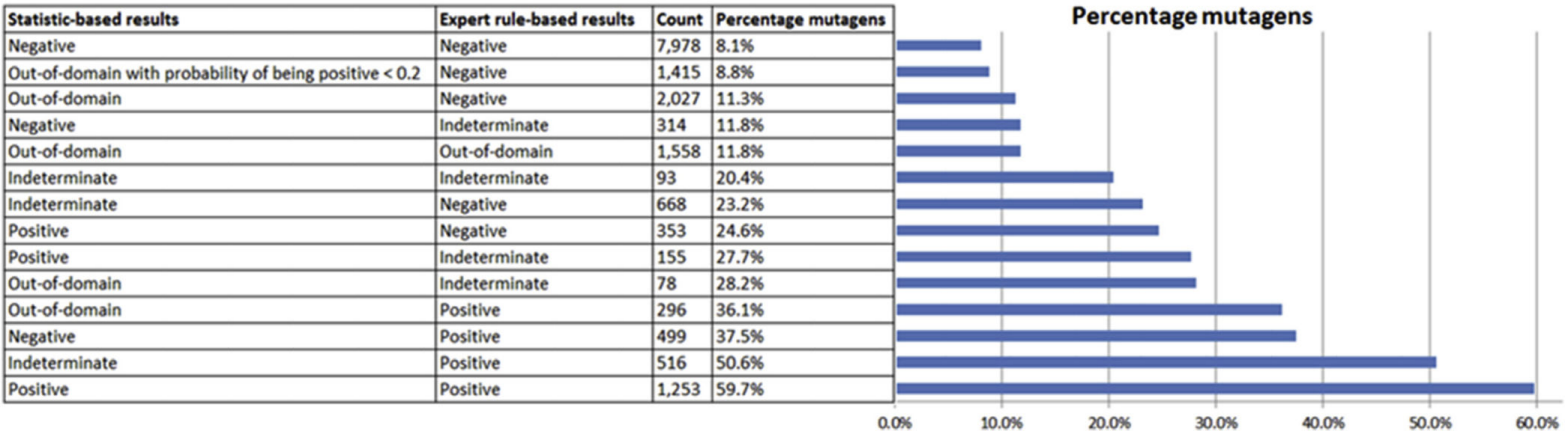
Summary of the likelihood of misclassifying a mutagenic impurity as non-mutagenic for different combinations of results.

A computational assessment of impurities should ideally balance the need for a rapid analysis of multiple compounds while limiting false negative and false positive predictions as much as practically possible. The information in Figs. 8 and 9 can be used to support such an analysis of the likelihood of misclassifying a mutagenic impurity as non-mutagenic based on different combinations of (Q)SAR results. This is important since all follow-on activities may compromise the desired high throughput goals if they are not tied to an assessment of the overall risk. A detailed expert review of out-of-domain and indeterminate results has time and cost implications, since it may require the gathering of a group of cross-discipline experts, performing literature searches, and/or instigating an additional analysis (such as a legal review) in order to reveal analogs that were previously designated as proprietary. On the other hand, assuming all out-of-domain or indeterminate results are potentially mutagenic has almost certainly greater time and cost implications, such as the need to perform additional laboratory test(s) (as well as the possible synthesis of the impurity) and/or implementation of control strategies. Hence, the level of any additional analysis, such as the extent of an expert review, should ideally take into consideration the likelihood of misclassifying a mutagenic impurity as non-mutagenic (i.e., a false negative prediction).

As discussed previously, there are a variety of approaches to resolve out-of-domain results. For example, as part of an expert review additional supportive evidence may be provided, including suitable analogs, an analysis of the lack of reactive potential as well as running another model (as discussed earlier). The likelihood of misclassifying a mutagenic impurity as non-mutagenic when one of the methodologies generates an out-of-domain result is in large part dependent on the result from the other model. Based on the analysis shown in Fig. 9, if a statistical-based model is out-of-domain and an expert rule-based model is positive, 36.2% of compounds are shown to be positive, whereas if the statistical-based model is out-of-domain and the expert rule-based model is negative then only 11.3% of compounds are positive. When the (Q)SAR model presents a result that is indeterminate, it may be prudent to examine the basis for the indeterminate call and determine through an expert review whether it can be refuted for valid reason, as discussed in Amberg et al. (2016) (e.g. a shared alert with known negative [ICH M7 class 4], an explanation based on the mechanism, an assessment of the relevance of features or underlying data from statistical-based methodologies, expert reviews based on chemical analogs from public or in-house sources, a visual inspection by an expert or an assessment of the strength of the single prediction).

Fig. 8 summarizes the frequency for the different (Q)SAR combinations and Fig. 9 the percentage of results that were experimentally identified Ames mutagens. The charts show the most common scenario is when the two systems predict the chemicals as a clear negative. The proportion of experimentally determined mutagens in this situation is similar to the pre-expert review performance (94%) reported in Dobo et al. (2012) and also in the same range as the reproducibility of the assay (McCann et al., 1984; Jolly et al., 2015). The analysis also shows that when the statistical-based model is negative (within the applicability domain of the model) and the expert rule-based model is indeterminate, then the likelihood of misclassifying a mutagenic impurity is also similar to two clear negatives (i.e., 11.8% vs. 8.1%). In addition, one of the most common scenarios is when a statistical-based model is out-of-domain and an expert rule-based model is negative (in domain). A subset of these examples is shown where the calculated probability of being positive from the statistical-based model is less than 0.2. This subset represents 1415 cases where the percentage of experimental mutagens is close to the case where both methodologies are clear negative. An expert review based on the low confidence or probability score alongside an assessment consistent with a clear negative, as discussed in Amberg et al. (2016), may be appropriate (i.e., “a rapid visual inspection of the results by the expert can be used to verify that no valid alerts for mutagenicity with a plausible mechanism were overlooked by the two (Q)SAR methodologies”).

It is also interesting to note that when the statistical-based model is out-of-domain and the expert rule-based model is positive then the percentage of Ames mutagens is 36.1%, which is similar to the situation when the statistical-based model is negative and the expert rule-based model is positive (37.5%). This may indicate that many of the chemicals predicted to be out-of-domain by the statistical-based model are related to novel APIs lacking reactive features. It is also worth pointing out that when an expert rule-based result is indeterminate, then the results from the statistical-based model are correlated with the percentage of Ames positive. When the statistical-based result is negative and the expert rule-based result it indeterminate, the percentage of Ames positives is 11.8% whereas when the statistical-based result is positive the percentage of Ames positives is 27.7% which illustrates the value of using the two complementary methodologies.

There are a number of limitations with this type of cross-pharmaceutical company analysis of proprietary data. It is not possible to see the individual chemicals to verify that there are no duplicate chemicals; however, this number will be low since different proprietary collections generally cover separate areas of the chemical space. The analysis is also based on a harmonized evaluation of different models and algorithms. In addition, the overall proportion of positive compounds differs across the individual proprietary collections; however, the general analysis is based on chemicals similar to APIs as well as chemicals typically used in chemical synthesis. Finally, there may be bias introduced since historical testing of impurities is generally performed on only class 3.

## 5. Summary and conclusion

As part of any ICH M7 analysis, an essential step is to combine the results from the two (Q)SAR methodologies consistent with the language in the ICH M7 guideline (i.e., “absence of structural alerts”). Out-of-domain results present particular challenges as to how this overall prediction should be generated. The underlying basis for this applicability domain analysis is often based upon a structural assessment, using similarity measures or an analysis of unknown fragments, potentially in combination with other information. The results from such applicability domain analysis may be presented as either inside or outside the applicability domain. However, additional information may be taken into consideration as part of an expert review that includes a weight-of-evidence re-assessment of the applicability domain, including relevant non-mutagenic analogs or additional model output (e.g., the confidence score or a calculated probability score of less than 0.2). The likelihood of misclassifying a mutagenic impurity as non-mutagenic in situations with out-of-domain calls when no alerts or other positive/indeterminate signals are identified is similar to when there are two clear negative results based on the analysis in this paper.

The current paper has reviewed different ways of handling out-of-domain and indeterminate results, including how to generate an overall prediction and any follow-up expert review or additional analysis (including an expert review based on analogs or an assessment of reactive features as well as the option of running another model) that is considered adequate based on the likelihood of misclassifying a mutagenic impurity. Over time, models that are routinely updated with the latest information, including the incorporation of knowledge from propriezry databases whenever possible, will reduce the number of false positives and false negatives, as well as out-of-domain and indeterminate results, further streamlining the ICH M7 (Q)SAR assessment process.

## Supplementary Material

1

2

## Figures and Tables

**Table 1:** The ICH M7 hazard classifications.

Class	Definition
1	Known mutagenic carcinogens
2	Known mutagens with unknown carcinogenic potential (bacterial mutagenicity positive,^a^ no rodent carcinogenicity data)
3	Alerting structure unrelated to the structure of the drug substance, no mutagenicity data
4	Alerting structure, same alert in drug substance or compounds related to the drug substance (e.g. process intermediates) which have been tested and are non-mutagenic
5	No structural alerts, or alerting structure with sufficient data to demonstrate lack of mutagenicity or carcinogenicity

a Or other relevant positive mutagenicity data indicative of DNA-reactivity-related induction of gene mutations (e.g., positive findings in *in vitro* gene mutation studies).

**Table 2:** Fields to analyze as part of the request to pharmaceutical companies.

Field	Description
Compound	A unique number to reference the compound but nothing to identify the chemical structure^a^.
Experimental Ames result	Include 1 for clear mutagens, 0 for clear non-mutagens. Do not include equivocal results.
Ames test description	A short description of how the Ames test was performed (e.g. GLP OECD 471/ICH S2 Ames).
Statistical-based model result	The prediction results and/or indication of whether it is out-of-domain or indeterminate.
Probability or other score	Any additional output, such as the probability of a positive outcome.
Rule-based (structural alert) result	The prediction result and/or indication of whether it is out-of-domain or indeterminate.
Precision or other score	Any additional output, such as the precision of the alert.

a This column may be blank.

**Table 3:** Summary of in domain predictions generated for the two (Q)SAR methodologies.

Statistic-based result	Expert rule-based result	Count^a^	Percentage of results that were experimentally identified Ames mutagens
Positive	Positive	1253	59.7%
Negative	Positive	499	37.5%
Positive	Negative	353	24.7%
Negative	Negative	7978	8.1%

a Out of 15,886 compounds tested.

**Table 4:** Summary of analysis where at least one of the methods generates an out-of-domain result.

Statistic-based result	Expert rule-based result	Count	Percentage of results that were experimentally identified Ames mutagens
Out-of-domain	Positive	296	36.2%
Out-of-domain	Indeterminate	78	28.2%
Out-of-domain	Out-of-domain	1558	11.8%
Out-of-domain	Negative	2027	11.8%

**Table 5:** Summary showing the effects of the confidence scores.

Statistic-based result	Expert rule-based result	Count	Percentage of results that were experimentally identified Ames mutagens
Out-of-domain with probability of being positive 0.2–0.4	Negative	339	17.1%
Out-of-domain with probability of being positive <0.2	Negative	1415	8.8%

**Table 6:** Different scenarios that include an indeterminate call from one or both of the methodologies.

Statistic-based result	Expert rule-based result	Count	Percentage of results that were experimentally identified Ames mutagens
Indeterminate	Positive	516	50.6%
Out-of-domain	Indeterminate	78	28.2%
Positive	Indeterminate	155	27.7%
Indeterminate	Negative	668	23.2%
Indeterminate	Indeterminate	93	20.4%
Negative	Indeterminate	314	11.8%

## Footnotes

2 The term “(Q)SAR” refers to (Quantitative) Structure-Activity Relationship and is used as an acronym for computational models that predict a biological response (such as mutagenicity) based on the chemical structure of the test molecule. The term collectively refers to both quantitative and non-quantitative (e.g., expert rule-based) structure-activity relationships by placing the “Q” in parentheses.
