# Supplementary material for: Principles and procedures for handling out-of-domain and indeterminate results as part of ICH M7 recommended (Q)SAR analyses
Source: Regul Toxicol Pharmacol. Author manuscript; Available in PMC 2020 Sep 18. (PMC7500704; doi:10.1016/j.yrtph.2018.12.007)
Supplement: 1 [file NIHMS1517744-supplement-1.docx]

**Supplemental information**

**Table S1: Rules for consolidating model outputs across different proprietary collections**

| **(Q)SAR Outcome** | **Methodology** | **System** | **Parameters** |
| --- | --- | --- | --- |
| Positive | Expert rule-based | Leadscope Genetox Expert Alerts | When Bacterial Mutation QSAR-only Prediction is Positive. |
| Positive | Statistical-based | Leadscope Genetox Statistical Suite | When Salmonella Mut QSAR-only Prediction* is Positive (within the applicability domain of the model) based on the setting that Salmonella Mut Positive Prediction Probability is greater than 0.6. |
| Positive | Expert rule-based | Derek Nexus | Probable, plausible and equivocal results . |
| Positive | Statistical-based | Sarah Nexus | Positive prediction. |
| Negative | Expert rule-based | Leadscope Genetox Expert Alerts | When Bacterial Mutation QSAR-only Prediction is Negative. |
| Negative | Statistical-based | Leadscope Genetox Statistical Suite | When Salmonella Mut QSAR-only Prediction* is Negative (within the applicability domain of the model) based on the setting that Salmonella Mut Positive Prediction Probability is less than 0.4. |
| Negative | Expert rule-based | Derek Nexus | The results are inactive. |
| Negative | Statistical-based | Sarah Nexus | No alert was detected, molecule was fully covered in the system. |
| Out-of-domain | Expert rule-based | Leadscope Genetox Expert Alerts | When Bacterial Mutation QSAR-only Prediction is Not In Domain. |
| Out-of-domain | Statistical-based | Leadscope Genetox Statistical Suite | When Salmonella Mut QSAR-only Prediction* is Not In Domain. |
| Out-of-domain | Statistical-based | Sarah Nexus | One or more unknown fragments were detected, generating an “outside domain” result. |
| Indeterminate | Expert rule-based | Leadscope Genetox Expert Alerts | When Bacterial Mutation QSAR-only Prediction** is Indeterminate. |
| Indeterminate | Statistical-based | Leadscope Genetox Statistical Suite | When Salmonella Mut QSAR-only Prediction* is Indeterminate (within the applicability domain of the model) based on the setting that Salmonella Mut Positive Prediction Probability is between than 0.4 and 0.6. |
| Indeterminate | Statistical-based | Sarah Nexus | An equivocal result was issued based on positive and potentially negative prediction hypotheses from the system or a lack of strong evidence to draw a conclusion. |
| Out-of-domain with probability of being positive < 0.2 | Statistical-based | Leadscope Genetox Statistical Suite | When Salmonella Mut QSAR-only Prediction** is Not In Domain and the Salmonella Mut Positive Prediction Probability is less than 0.2. |
| Out-of-domain with probability of being positive 0.2 – 0.4 | Statistical-based | Leadscope Genetox Statistical Suite | When Salmonella Mut QSAR-only Prediction** is Not In Domain and the Salmonella Mut Positive Prediction Probability is between than 0.2 and 0.4. |

Notes: * The Leadscope Salmonella Mut v3 was used for this assessment. The Leadscope E.Coli/TA102 model is generally used in combination with the Salmonella mut model; however, an out-of-domain E.Coli/TA102 result would not overrule a Salmonella Mut in domain result based on the Ames consensus rules (Leadscope 2018).** It should be noted that only Leadscope predictions generated a probability that could be used to assess “Out-of-domain with probability of being positive < 0.2” and “Out-of-domain with probability of being positive 0.2-0.4” categories.
